# An algorithm to reduce human–robot interface compliance errors in posture estimation in wearable robots

**DOI:** 10.1017/wtc.2022.29

**Published:** 2022-12-27

**Authors:** Gleb Koginov, Kanako Sternberg, Peter Wolf, Kai Schmidt, Jaime E. Duarte, Robert Riener

**Affiliations:** 1 Sensory-Motor Systems Lab, Institute of Robotics and Intelligent Systems, Zürich, Switzerland; 2 MyoSwiss AG, Zürich, Switzerland; 3 Reharobotics Group, Spinal Cord Injury Center, Balgrist University Hospital, Medical Faculty, University of Zurich, Zürich, Switzerland

**Keywords:** control, exoskeletons, exosuits, intelligent orthotics, rehabilitation robotics

## Abstract

Assistive forces transmitted from wearable robots to the robot’s users are often defined by controllers that rely on the accurate estimation of the human posture. The compliant nature of the human–robot interface can negatively affect the robot’s ability to estimate the posture. In this article, we present a novel algorithm that uses machine learning to correct these errors in posture estimation. For that, we recorded motion capture data and robot performance data from a group of participants (*n* = 8; 4 females) who walked on a treadmill while wearing a wearable robot, the Myosuit. Participants walked on level ground at various gait speeds and levels of support from the Myosuit. We used optical motion capture data to measure the relative displacement between the person and the Myosuit. We then combined this data with data derived from the robot to train a model, using a grading boosting algorithm (XGBoost), that corrected for the mechanical compliance errors in posture estimation. For the Myosuit controller, we were particularly interested in the angle of the thigh segment. Using our algorithm, the estimated thigh segment’s angle RMS error was reduced from 6.3° (2.3°) to 2.5° (1.0°), mean (standard deviation). The average maximum error was reduced from 13.1° (4.9°) to 5.9° (2.1°). These improvements in posture estimation were observed for all of the considered assistance force levels and walking speeds. This suggests that ML-based algorithms provide a promising opportunity to be used in combination with wearable-robot sensors for an accurate user posture estimation.

## 1. Introduction

The field of wearable robots has seen major developments over the past decade. These devices have been shown to make strenuous tasks easier (Mooney et al., 2014; Awad et al., 2017; Seo et al., 2017), provide compensation for gait impairments (Lerner et al., 2017, 2018; Awad et al., 2020; Haufe et al., 2020), and partially compensate for muscle weakness effects due to aging (Martini et al., 2019). A common issue faced by the field is the difficulty to accurately estimate the orientation of the wearer’s limb segments, which is crucial for the control of a wearable robot (Vu et al., 2020; Haque et al., 2021). In various applications of wearable robots, hip (Jang et al., 2016; Tanghe et al., 2016), knee (Schmidt et al., 2017), and ankle (van Dijk et al., 2017; Baud et al., 2021; Xiloyannis et al., 2021) joint angles have been used as inputs to the robot’s controllers to identify gait events and classify the activity done by the user. The accuracy of estimation is typically affected by two sources of error: (1) the mapping of readings from sensors like accelerometers and gyroscopes to an orientation (e.g., Kalman filters), and (2) the soft (compliant) nature of the interface between the human and the robot. In this article, we present a method to correct the error in posture estimation due to compliance.

Compliance, defined here as the relative motion between the robot and its wearer, is common in wearable robots which transmit forces from the robot’s structure to the human’s skeleton through a physical human–robot interface. This human–robot interface typically includes some type of padding on the robot’s structure, the human’s clothing (if not worn directly on the skin), and the human’s soft tissue – a combination of skin, muscle, and fat – before reaching the human’s skeleton. Compliance is a complex issue and can have varying magnitude depending on the amount and the timing of the applied assistance, dynamics of the performed movement, and the orientation of the human’s limbs. Its underlying causes depend on a set of finite factors:

1. Time-dependent slippage (i.e system settling) of the robot on the human. For example, the system settles over time into an energetically favorable position on the human body.
2. Stiffness of the human–robot interface at the site of force anchoring and force application. The compliance phenomenon depends, among others, on the stiffness of the human tissue and how tight the robot is strapped to the user.
3. Misalignment of the biological and the robotic joints. Depending on the robot’s design, the instantaneous centers of rotation of the biological joints and the corresponding robotic joints may not match.
4. Compliance of the robot’s own structures. Exoskeletons may use rigid structures with joint-aligned drives or softer structures that transmit forces with compliant tendons. The robot’s structure deforms with the applied force to a degree that depends on the exact design.

In a lab environment, where camera-based motion capture systems can be used to measure a person’s posture, the errors in posture estimation due to compliance can be minimized by measuring the person’s limb segments directly. Outside of the lab environment, inertial sensors (IMUs) are frequently used to estimate the user’s posture due to their cost-effectiveness and miniature size (Caldas et al., 2017; Lee et al., 2021). These sensors are typically mounted on the robot’s structure rather than directly on the user’s limbs. As a result, the sensors measure the robot’s movements which are then used as a proxy for the user’s posture. Depending on the compliance of the human–robot interface, the user’s posture estimation can be negatively affected. This is because any displacement of the robot’s structure relative to the human body is reflected in the readings of the sensors. Haque et al. proposed a design of a passive exoskeleton structure to mount a series of sensors (including IMUs) to estimate the lower limb segment angles (Haque et al., 2021). Their results showed that, for both knee and ankle joints, deviations of up to 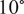 happen at different phases of a gait cycle, partly attributed to the relative motion between the human and the exoskeleton. As the proposed device did not actively apply forces to the human body, one can expect that errors in posture estimation are even larger in active systems. Such deviations are particularly noticeable in the control strategies that scale the assistive force based on the gait symmetry, as proposed by Malcolm et al. (2018) and Aguirre-Ollinger and Yu (2021).

One approach to improve posture estimation is the use of machine learning (ML) tools. ML, or pattern recognition methods, have been successfully used for many gait analysis problems, including the classification of activity type (Bhakta et al., 2020), estimation of ambulation speed (Zhang et al., 2020), estimation of user’s joint angles (Mundt et al., 2020), estimation of a biological joint torque (Molinaro et al., 2020), and classification of gait phases (Yang et al., 2019). The methods implemented range from simple (e.g., linear discriminant analysis) to complex (artificial neural networks). The simpler methods are generally easier to use and require less training data. However, their generalizability to various activities and users is limited. For real-world applications, the ability to generalize to a wide range of the population in a user-independent manner is crucial. Additionally, the estimation algorithm should not be limited to a single activity (i.e., walking at constant speed) or assistance level (the robot does not apply the same level of assistance for all users and tasks). A more applicable methodology should instead perform the estimation independent of the user, for a range of walking speeds, and for different levels of assistance. Depending on the intended use, an ability to correct the segment compliance errors in an online fashion with minimal delay may also be required. More complex models often offer a larger solution space. However, such models often require larger data sets, more powerful computational hardware, and a longer training process, all of which may not be feasible depending on the type of the problem considered.

In this article, we present a novel algorithm that improves the orientation estimation of individual limb segments (combination of which describes a user’s *posture*) of a wearable robot’s user. We show that the algorithm is capable of achieving this result in real time and in a user-independent manner. The algorithm corrects for errors in the estimation of limb segment angles that arise due to the compliance that exists between a wearable robot and its user. To develop the algorithm, we first designed a protocol to measure the relative motion between the structure of a walking assistance robot (Myosuit by MyoSwiss AG, Switzerland) and the biological segments (thigh and shank) of its wearer. The protocol required participants to walk at various speeds and at various settings of Myosuit’s assistance level. The assistance level was varied as the past literature has shown that the loading of the human–robot interface correlated to the relative displacement between the human and the robot (Langlois et al., 2021). We then used a gradient boosting algorithm, XGBoost (Chen and Guestrin, 2016), to account for the variability in compliance that comes from various robot designs, device configurations, and users’ bodies. Finally, we showed that, for the subjects not in the training data set, the error between the human segment angles and the ML algorithm estimated angles was smaller than the error between the human segment angles and the robot segment angles. Our algorithm can help improve the estimation of lower-limb kinematics by wearable robots. This can improve the performance of controllers and ultimately lead to a better delivery of assistance from the robot to the user.

## 2. Methodology

### 2.1. Errors in posture estimation using robot-mounted sensors

Wearable robots use various types of sensors to estimate the wearer’s posture as an input to their controllers. Here, we refer to a human’s posture as a set of limb segment angles (e.g., thigh or shank).

When a limb segment’s angle is estimated by a sensor mounted directly on that segment, the resultant measurement can be formulated as
(1)

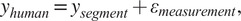

where 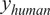 is the measurement output from a sensor mounted directly on the human segment; 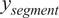 is the true segment angle; 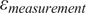 is the estimation error due to the quality of the sensor’s readings and the algorithm used to estimate the angle (e.g., Kalman filter).

If the sensor is instead mounted on a structure, as is the case in wearable robots, and this structure is mounted on the limb segment, the sensor measurement output is affected by an additional source of error:
(2)

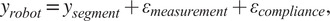

where 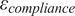 is the estimation error due to the compliance of the human–robot interface.

Considering the two types of errors, we know that 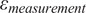 error is dependent on the type of the sensor being used, the environmental conditions in which that sensor is used, and on the algorithm for the estimation of the posture based on the sensor’s raw data. Because this is a common problem for many of motion-tracking systems and applications, much literature has been previously devoted to the problem of modeling the 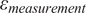 term.

For our work, we focused on quantifying the error due to the 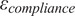 term. To isolate it from the 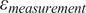 term, we used a state-of-the-art camera-based motion capture system (Vicon, Oxford, UK) to simultaneously measure both the human’s limb segment angles (i.e., 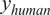 ) and the corresponding robotic segment angle (i.e., 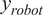 ). We then derived the assumption that because both 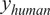 and 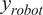 were measured simultaneously and by the same measurement system, their corresponding measurement errors could be canceled out to define the error due to compliance as
(3)

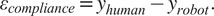



To capture the nonlinear behavior of the compliance effects, we have encoded the 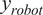 term as well as other robot-derived sensor signals in a matrix 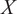 (more details on the list of used signals can be found in Table 1) and have defined a mapping in the form
(4)

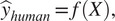

where 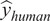 is the estimated human-segment angle, corrected for the compliance effects, and 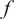 is a mapping function.

**Table 1.** List of features used for the algorithm

Signal	Symbol	Unit	Group	Data source
Robot segment angles	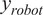	deg	Robot performance data	Motion capture
Right motor current	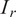	mA	Robot performance data	Robot
Left leg state (stance or swing)	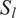	—	State data	Robot
Right leg state (stance or swing)	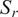	—	State data	Robot
Chosen mode of assistance	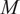	—	Robot performance data	Robot
Assistance level selected	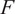	—	Robot performance data	Robot
*X*, *Y*, *Z* axes of gyroscope	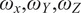	mdeg/s	Human motion data	Robot
*X*, *Y*, *Z* axes of accelerometer	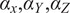	m/s^2^	Human motion data	Robot
Right motor hall sensor counts	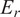	count	Human motion data	Robot

*Note. The signal units reported in the table show the convention used as an input into the algorithm.*

We modeled the 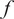 mapping function using machine learning to show that
(5)

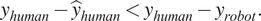



Throughout this project, we use the following naming convention:

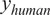
 – human segment angles: These angles were derived from a marker point cloud with a motion capture system and represent the target variable in our algorithm.

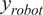
 – robot segment angles: These angles were derived from a marker point cloud with a motion capture system. The values imitate segment angle measurements of a robot. The values of 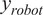 were used as one of the features in our algorithm.

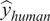
 – estimated human segment angles: These angles represent the estimation of the target variable by our algorithm using 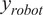 and robot-derived sensor measurements (see Table 1 for more details) as input features.

### 2.2. Participant recruitment

Eight unimpaired participants (4 female; height: 1.72 (1.62–1.95) m; mass: 63.77 (51–85) kg, mean (range)) were recruited for this study through word of mouth. The study protocol was reviewed and approved by the institutional board of ETH Zurich, Switzerland (reference number: EK 2019-N-119). All participants provided written informed consent for their participation in the experiment. Four out of the eight participants had previous experience wearing a wearable robot for the lower limbs. The previous experience had no impact on the results of this study as previous experience with a wearable robot does not affect the compliance of the robot-human interface.

### 2.3. Wearable robot

**Figure 1.**
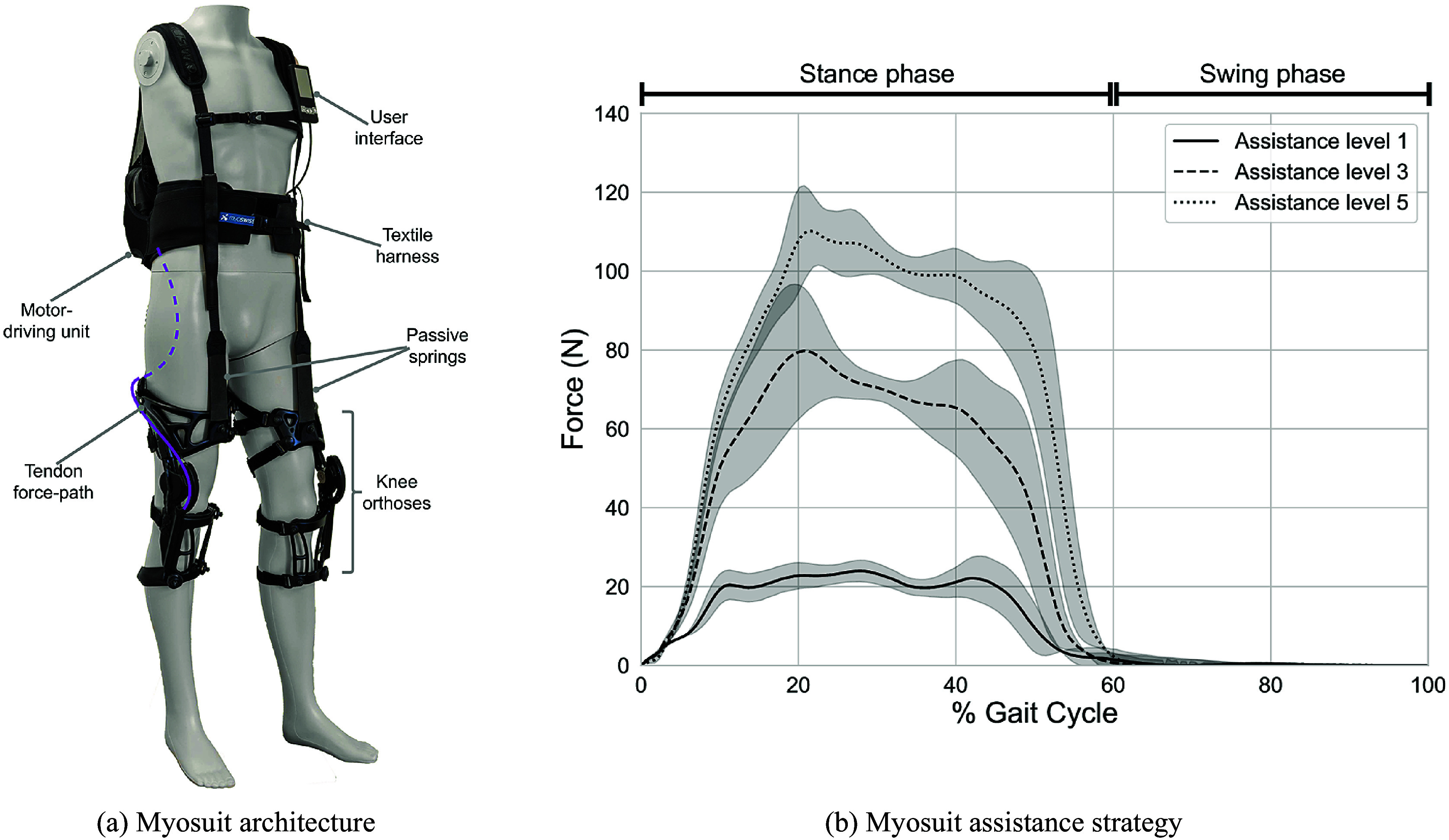
Architecture and the operation principle of the Myosuit. (a) The Myosuit is a textile-based wearable robot to support the lower limbs. It is comprised of a textile harness that houses two motors, control electronics, and a battery. Two artificial tendons are routed from the motors posteriorly over the hip joint and anteriorly over the knee joint. Low-weight orthoses are placed on the user’s lower limbs to route and anchor the tendons. (b) The Myosuit supports the weight-bearing phase of walking. Here the mean and standard deviation of the forces measured during the experimental protocol and averaged across all participants and conditions are shown. The assisting forces are modulated based on the relative angle between the thigh and shank segments. The segment angles and walking events are estimated using a set of 9-axis IMUs mounted on the shank, thigh, and trunk segments of the user’s body.

We used the Myosuit (MyoSwiss AG, Zurich, Switzerland) as an example of a wearable robot. The Myosuit was designed to support a person’s weight-bearing capacity during activities of daily life that include walking and standing (Haufe et al., 2021). The full system weighs 5.5 kg. The device (Figure 1a) includes a backpack-like motor-driving unit that houses two motor-gearbox-pulley assemblies (one per leg), control electronics, and one Li-Ion battery pack. Two hard-shell plastic knee orthoses are placed on each leg to route an artificial tendon and anchor forces along the leg. Each leg is supported by an ultra-high-molecular-weight polyethylene cable routed posteriorly over the hip joint, laterally over the thigh, and anteriorly over the knee joint of the orthosis, anchoring at its distal shank component. Two passive polymer springs span the front of the hip joint. The springs were tensioned just enough to counteract potential downward slipping of the knee orthoses. The Myosuit includes five Inertial Measurement Units (IMUs); two on each thigh and shank segments and the fifth one in the motor-driving unit. A combination of the IMU signals is used to calculate the posture of the user’s five-segment body model at 100 Hz. The relative knee joint angle is used as an input for the instantaneous modulation of the assistance force (Figure 1b). The relative hip joint angle, as well as the raw IMU sensor signals, are used for the detection of key gait events.

When used for overground walking, the peak linear force of that can be applied through the tendons during the stance phase was 130 N. This force supports the extension of the knee and hip joints. The onset of the Myosuit’s support is right after detecting the user’s heel strike. The Myosuit provides no active assistance during the swing phase of the gait cycle. The switch between the stance and swing states happens at around 40–45% of the gait cycle–toward the end of the weight-bearing period of the stance phase (Figure 1b). The peak support force can be adjusted over 6 levels (0 to 5) between 0 N and 130 N (e.g., assistance level 3/5 means 60% of the peak 130 N force is available during the active support phase).

The Myosuit was donned on the participants according to its user manual. More details on the architecture of the device can be found in prior literature (Haufe et al., 2020).

### 2.4. Experimental protocol

**Figure 2.**
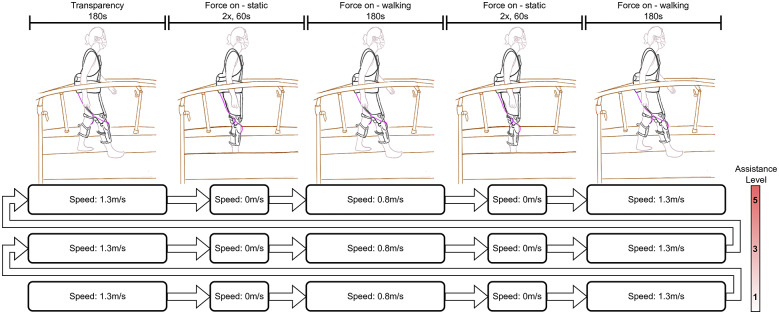
Graphical representation of the study design. The participants were asked to walk at three levels of Myosuit assistance. For each of these levels, the participants walked in transparency mode at 0.8, and 1.3 m/s with Myosuit assistance turned on. In between each of these dynamic conditions, a static force ramping experiment was performed. For that, the participants were asked to stand still and a target force of 130 N was applied twice. The overall duration of the experiment was approximately 90 min, including the time for Myosuit donning and familiarization.

Each participant completed three experimental blocks in a single session. For the experiment, participants walked on a split-belt treadmill (V-Gait Dual Belt, Motekforce Link, Netherlands) while wearing the Myosuit (see Figure 2). The session lasted approximately 90 min, including the time for donning and familiarization of the Myosuit.

In each experimental condition, participants first walked for 3 min in a “zero-force” (i.e., transparent) condition. Here, the Myosuit was set to simply modulate the cable length such that the tissue compliance, limb configuration, and joint angular velocity were compensated for. After walking for 3 min, the participants were asked to stand still and a constant force of 130 N was applied by the Myosuit twice following a ramp input, for a total duration of 1 min. Participants then walked for 3 min at a speed of 0.8 m/s (constant speed controlled by the treadmill) with the Myosuit’s assistance turned on. Subsequently, the participants were asked to stand still and the constant force of 130 N was again applied twice for a total duration of 1 min. Finally, the participants walked for 3 min at a speed of 1.3 m/s (constant speed controlled by the treadmill) with the Myosuit’s assistance turned on. The assistance level was increased between the experimental blocks from levels 1 (maximum assistance 25 N) to 3 (maximum assistance 75 N) and 5 (maximum assistance 130 N).

### 2.5. Data acquisition

The kinematics of the right leg of the human limb segments and robot braces were measured using a camera-based motion capture system. For the human limb segments, two clouds of markers were placed directly on the soft tissue of the participants’ thigh (four markers) and shank (five markers). For the robot, two clouds of four markers were placed on the thigh and shank parts of the knee orthosis. Whenever possible, the markers were placed in an orthogonal configuration as suggested by Söderkvist and Wedin (1993). To minimize the risk of marker occlusion by the robot’s components, some markers were raised from their base using 3D-printed pillars. The markers were placed only on the right side because of the setup symmetry. We focus our analysis on the thigh and shank segments only because these are the main segments of interest for Myosuit’s control algorithm.

**Figure 3.**
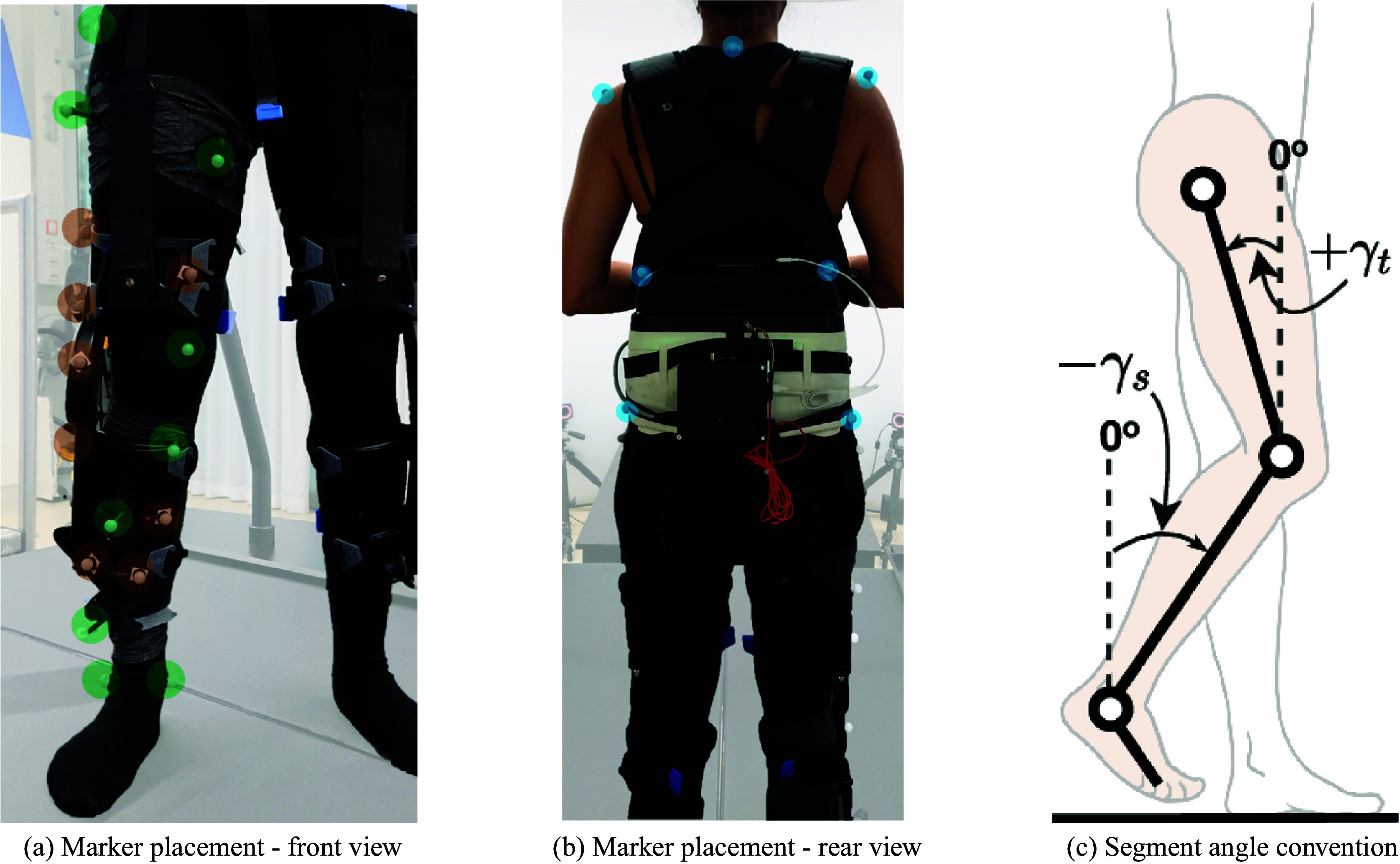
Marker placements from the front (a) and rear (b). Clouds of four and five markers were placed on the participant’s thigh and shank, respectively (highlighted in green). Clouds of four markers were placed on the thigh and shank components of the Myosuit (highlighted in orange). The choice of marker cloud sizes was driven by the initial sensitivity study where the chance of occlusion, marker loss, and marker stability were analysed. Additionally, markers were placed on the motor driving unit, left and right acromion, and the c7 vertebrae (highlighted in blue). (c) Angle convention for the shank and thigh segments in sagittal plane. The thigh angle (here 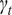 ) is measured between the biological thigh and a vertical line passing through the knee joint’s centerline, with positive angles measured in the counter-clockwise direction. The shank angle (here 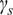 ) is measured between the biological shank and the vertical line passing through the ankle joint’s centerline, with positive angles measured in the counter-clockwise direction. This angular convention was chosen as it matched the one used by the Myosuit controller.

To assist in the post-processing, seven additional markers were tracked (four on the motor-driving unit, two on the left and right acromion, and one on the C7 vertebrae). The total marker set during the experiment consisted of 25 markers (see Figure 3a,b).

The tension on the right tendon of the robot was measured with a load-cell (Miniature S-Beam FSH04416, Futek Advanced Sensor Technology, USA) placed proximally at the output of the motor-driving unit. For that, upon leaving the motor-gearbox-pulley assembly, the tendon was routed over a miniature pulley mounted on top of the S-Beam load-cell. The load-cell signals were used only in the data post-processing to confirm that the system was functioning appropriately (i.e., applying the forces as expected). Considering the limited scope of the load-cell’s purpose and the negligible friction between the tendon and the pulley mechanism, a uni-axial load-cell was used.

The position of the markers was recorded at 100 Hz using an array of 10 cameras (Bonita B10, VICON, UK). The sensor signals measured by the Myosuit were logged at 100 Hz. Table 1 lists all the signals logged by the Myosuit.

### 2.6. Data processing

The data from the motion capture and the Myosuit were synchronized using an external trigger signal. The data were then interpolated to the same time axis for alignment. The resultant time series were split into gait cycles based on the stance and swing detection algorithm of the Myosuit. The data of the motion capture markers were first processed through the Vicon Nexus software to label each marker and correct the gaps in the measured trajectories. For each experiment, the first 5 seconds (500 frames) of the static condition were averaged to calculate the reference marker cloud:
(6)

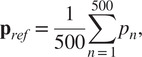

where 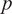 and 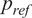 are 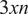 matrices of *n* marker points.

The rigid best-fit transformations from the reference marker cloud to each recorded frame were then calculated following the approach described by Sorkine-Hornung and Rabinovich (2017) with identity weight matrix. For that, we first define the rotation and translation transformation problem in least-squares form as
(7)

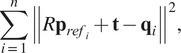



where 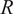 is the 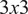 orthogonal matrix representing the cloud rotation, 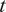 is the cloud translation vector, and 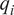 represents the individual markers of a particular point cloud frame. The calculation of the rotation matrix can be decoupled from the calculation of the translation by assuming (temporarily) the latter to be zero. We can then define matrices 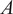 and 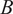 as 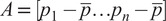 and 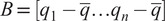 , where
(8)

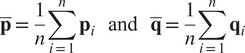



and their “covariance” matrix as
(9)

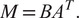



The singular value decomposition of the *M* matrix is given by
(10)

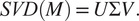



Finally, the rotation matrix can be calculated as
(11)

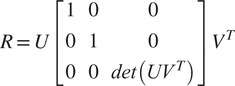



and substituted into (7) to find the translation as
(12)

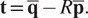



A local coordinate system was assigned to every reconstructed marker set. The vectors in this local coordinate system were used to calculate the angles of the segments in the sagittal plane relative to a vertical (see Figure 3c).

### 2.7. Pipeline for compliance error compensation

**Figure 4.**
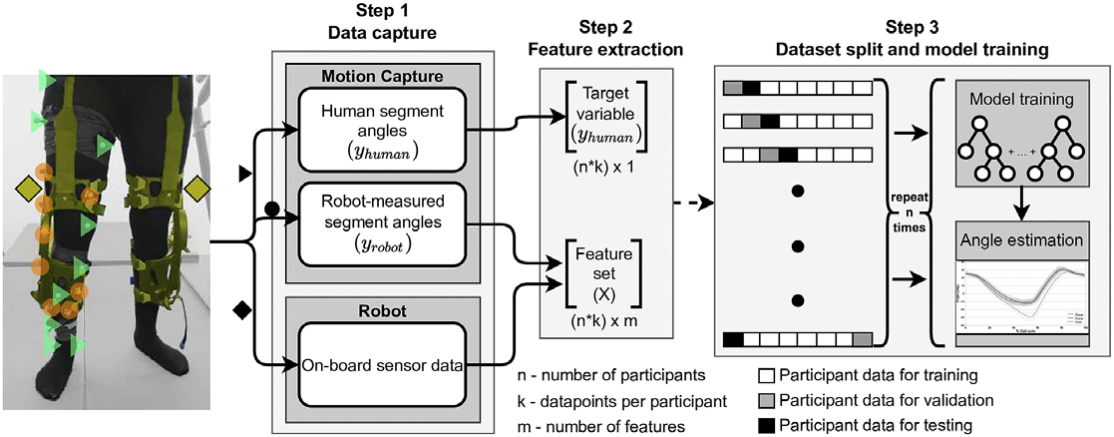
Schematic representation of the implemented pipeline for compliance error compensation. Three main sources of data are used: motion capture of human segments (triangles, 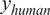 ) and robot segments (circles, 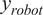 ) and robot-sensor derived data (rhombus). The latter and the 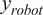 are used to construct the feature vector for the gradient boosting algorithm. The 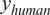 variable is used as the target variable. The data from the eight study participants are then arranged such that six participants are part of the training set, one is used for the validation set, and one for the model testing set. This splitting strategy was repeated eight times to show the model generalizability across the data of all of the study participants.

In this section we introduce the pipeline to create the model to compensate for compliance errors in segment angle estimation (see Figure 4) with the following steps:

1. Combine the *robot segment angles* ( 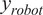 ) with the robot-derived data (see Table 1 for more details) to create a feature vector.
2. Set the motion capture data of the *human segment angles* ( 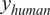 ) as the target vector.
3. Use a gradient boosting algorithm (XGBoost) to fit the regression model to the aforementioned data.
4. Use the trained regression model, together with the new feature vector, to calculate the *estimated human segment angles* ( 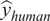 ).

Ultimately, the aim of the model is to reconstruct 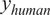 using only inputs from the robot. We limited the set of features fed to the algorithm to those that would be commonly available on a lower-limb wearable robot. We grouped these features into the following three categories: (1) robot performance data (e.g., applied motor torque or current), (2) state data (e.g., stance or swing state of the leg), and (3) human motion data (e.g., encoder counts or IMU data). We chose to not use windowing functions and only use the real-time signal data to ensure that no delay is introduced to the robot controllers when this model is used. The list of features, and their group assignments, that were used to train the regression model in this project are shown in Table 1.

We assessed the performance of the algorithm by looking at two key metrics: (1) root-mean-squared (RMS) compliance error and (2) peak angular compliance error, averaged over all gait cycles for a particular study participant.

To test the model’s generalizability, we split the data sets from the eight participants as follows:

- data from six participants to train the model,
- data from one participant to validate the model, and
- data from one participant to test the model.

The aim of this configuration was to ensure that none of the data of the participants used in the testing phase were part of either the training or validation data sets. This splitting strategy was repeated eight times, always leaving one participant’s data for validation and another participant’s data for testing (Figure 4). The training of the algorithm was done using the XGBoost module in Python 3.8. Following the preliminary analysis and using the values derived by Molinaro et al. (2020), the following parameters were used as shown in Table 2.

**Table 2.** List of tuned XGBoost hyperparameters used in the segment estimation algorithm

Hyperparameter	Symbol	Value
Learning rate	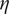	0.3
Max depth of a tree	—	10
Min loss reduction for a node split	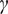	0
L2 regularization	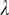	1
L1 regularization	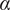	0

We chose to train separate models for the thigh and shank segments since each segment was expected to experience different compliance effects. This is due to factors like (1) interface stiffness, (2) force application areas, and (3) transmitted forces and their losses, among others. Because the implemented pipeline matches exactly for both thigh and shank, we focused our efforts on the segment with the larger error between the robot and the human segment angles.

### 2.8. Influence of force and speed on compliance

We performed statistical analysis to assess the correlation between the average RMS error and the force and speed parameters. For that, the conditional mean of the mean RMS error was computed with a linear predictor model taking into account speed, force, and their interaction. A random intercept variable in the form of subject id was added to the model to decouple the subject dependency. The implementation of the model was performed using *lmer* (*Linear Mixed-Effects Models*) package for R programming language.

## 3. Results

**Figure 5.**
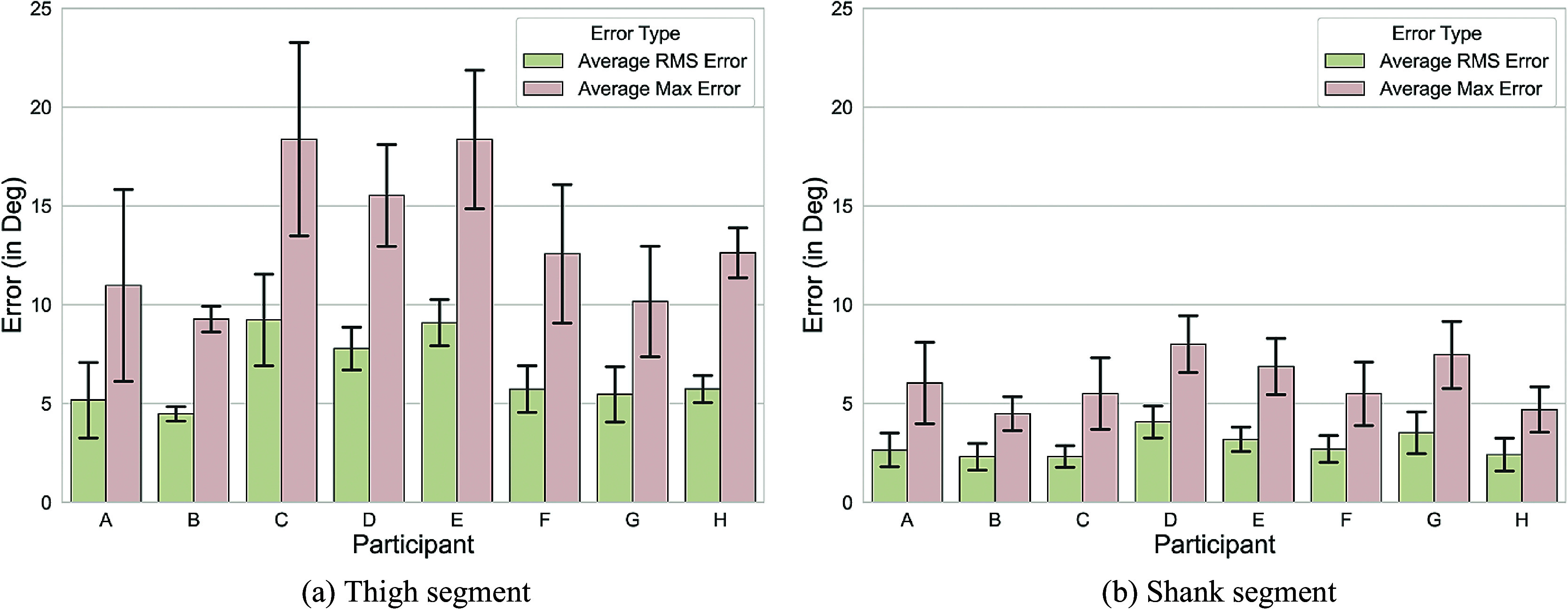
Compliance errors. ( 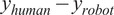 ) RMS errors for (a) thigh and (b) shank segments averaged across all gait cycles. The error bars represent ± 1 standard deviation.

When averaged over all experimental conditions, the average compliance-driven RMS errors between the *robot segment angles* and the *human segment angles* ( 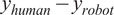 ) were higher for the thigh segment (6.4° (2.3°), mean (standard deviation, SD)) than the shank segment (2.7° (0.9°)) for all participants (see Figure 5a,b). Therefore, we focused our subsequent attention to the thigh segment only, noting that the methodology described in Section 2 is also valid for the shank segment.

**Figure 6.**
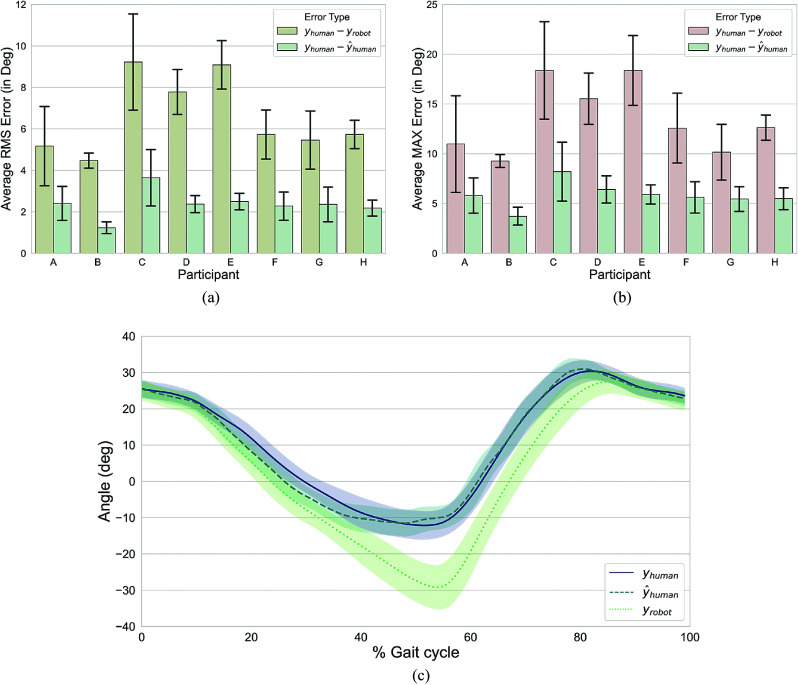
Model results. (a,b) Comparison of the thigh segment angle errors. The compliance errors before and after the correction by the XGBoost models are displayed. (a) The averaged RMS and (b) the averaged maximum angle errors. The error bars represent ± 1 standard deviation. (c) Thigh segment angle throughout the gait cycle for participant E. The plot shows mean and standard deviation over 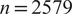 gait cycles averaged over all assistance and speed levels. The results of 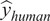 represent the performance of our algorithm tested on the data of participant E in a subject independent manner (i.e the particular participant’s data were not used for model training or validation).

We compared the participants’ segment angles obtained from our model to those measured by a motion capture system (monitoring a marker point cloud attached to the robot) (( 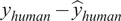 ) vs ( 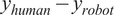 )). The model reduced the average RMS ( 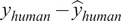 ) error to under 2.5° (1.0°) across all but one participant (see Figure 6a). Participant B had the lowest average RMS error of 1.2° (0.3°). Participant C had the largest average RMS error of 3.6° (1.4°). The model reduced the average peak error values over a gait cycle for the thigh segment with most participants showing a two-fold, or higher, reduction in error (see Figure 6b). Participant C had the highest average maximum error at 8.2°(3.0°).

Across the complete gait cycle, the compliance error was most prominent in the regions between 40% and 80% (see Figure 6c for a representative participant; participant E). Here, 0% gait cycle was defined as a time point of initial contact and 100% as being the toe-off event.

**Figure 7.**
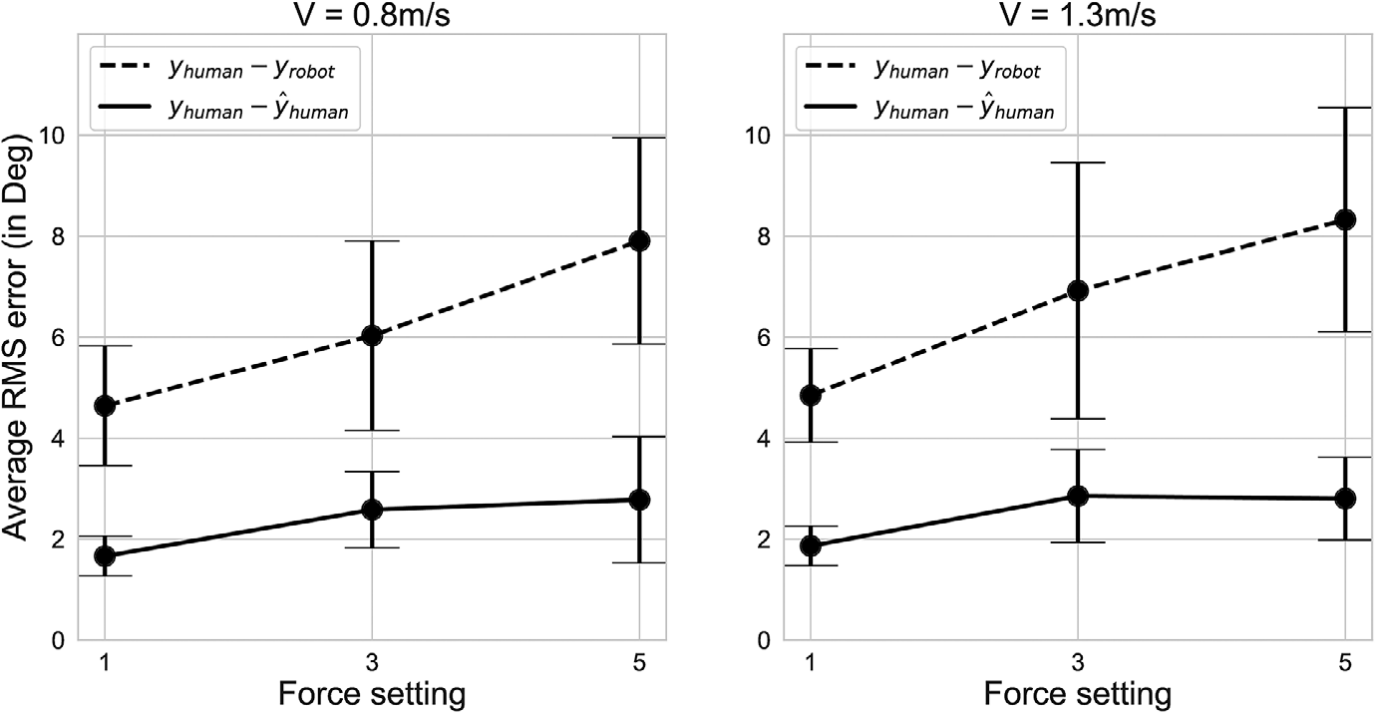
Effects of force and speed. Plots showing the dependencies of the corrected and the uncorrected mean RMS errors on the assistance level used. Results for both 0.8 and 1.3 m/s speeds are shown on the left and the right-hand sides, respectively. The error bars represent ± 1 standard deviation.

The largest compliance errors were observed at the highest force and walking speed condition, that is, at assistance level of 5 and walking speed of 1.3 m/s (see Figure 7). The standard deviation was smallest at the lowest walking speed and increased when the higher forces were used. No meaningful correlation between the two speed profiles and the compliance error were observed. A near-linear trend was found between the three chosen force levels and the magnitude of the mean RMS error (*F*(6.24) = 2.00, *p* < 0.001). For all the assistance levels, the errors were consistently lower after the measurements were corrected by our model.

## 4. Discussion

### 4.1. A machine learning algorithm improves posture estimation in wearable robots

The proposed ML algorithm improved the posture estimation in a wearable robot by compensating for posture estimation errors that are due to the compliance in the human–robot interface. For all but one participant the average RMS error over all gait cycles was under 2.5°. An almost three-fold error reduction was obtained for participant E where the average RMS error was reduced from 9.1° (1.2°) (mean (SD)) to 2.5° (0.4°), and the average maximum error was reduced from 18.4° (3.5°) to 5.9° (1.0°). The largest post-correction error was observed for participant C with an average RMS error of 3.6° (1.4°). Our results support the use of ML approaches in the domain of posture estimation where errors due to compliance can be partially negated with machine learning algorithms.

The proposed method has important real-world applications for wearable robots. First, by using the leave-one-out approach, we showed that our algorithm was able to generalize and reduce the compliance-driven posture estimation error for the participants of wide range of height (1.62–1.95 m) and mass (51–85 kg) and who were not part of the training data set. This is key to ensure that this approach can be applied to a wide range of users and robot configurations (Bhakta et al., 2020). Second, the results suggest that only a limited number of participants and features are needed to achieve similar levels of error reduction. This may be attributed, among others, to the fact that both training and testing data sets consisted of the same walking speed and assistance force conditions. The pool of participants used in our study (8 participants) is in line with other similar studies, ranging between 8 and 14 participants (Bhakta et al., 2020; Zhang et al., 2020). As the effects of compliance depend on both stiffness of the human–robot interface and the mode of the applied assistive forces, it is beneficial for the performance of the algorithm to capture both of these features by including participant of a wide variety of body types as well as varying the range of the robot-applied forces. Third, this method was able to estimate lower-limb segment angles without information about the robot’s fit on the user’s body or the user’s body parameters. This is important for real-world application, as measuring and entering such information is time consuming and, as such, would be unrealistic in, for example, a clinical setting. Finally, the proposed method can be implemented in a real-time environment as no information about the future data was encoded. Our proposed algorithm also did not rely on time-based windowing functions for feature calculations. While, as highlighted by Mundt et al. (2020), such a design of a feature tree may lead to noise in the segment angle estimation, it introduces no delay. Such behavior can be advantageous for the implementation in the control algorithms of wearable robotics, as it makes them inherently more responsive to the user’s inputs.

### 4.2. Compliance errors are largest in the second half of the gait cycle

The average angular deviation between human thigh and robot structure was small in the first 45% of gait cycle (see Figure 6c). The error between the true and robot segment angles started increasing from 45% and reached the peak around the 55% mark of the gait cycle. The average error then gradually reduced to a lower value until around 87% of the gait cycle. The development of the error matches with the timing of the Myosuit’s force application. The force was applied at initial contact (0% of the gait cycle) and was then modulated until the user reached the end of the weight-bearing phase (around 45% of the gait cycle) when the Myosuit transitioned to the zero-force mode. The low magnitude of the average error in the region between 0% and 45% of the gait cycle can be explained by the activation of the assistance force which increased the stiffness of the human–robot interface thus resulting in the system settling on the human body.

The sharp release of the forces at 40% of the gait cycle resulted in the relaxation of the human–robot interface and an increase in the compliance error. The increasing deviation in the angular estimation around the middle of the gait cycle could be attributed to the miss-alignment between the pivot points of the robotic structure and the biological knee joint. The relative orientation of the human segment and the robotic segment then stayed constant until the period of terminal swing. The reduction of the relative error in the terminal swing region may be attributed to the Myosuit aligning better with the user’s thigh segment at that particular configuration.

The individual limb segment angles are used by the controller of the Myosuit for both the active assistance phase (between 0% and 45% of the gait cycle) and the transparency phase (between 45% and 100% of the gait cycle).

In the active phase, the Myosuit uses a polynomial mapping between the thigh and the shank segment angles, and the target applied assistive force. A compliance-driven error in this phase can thus lead to a deviation from the designed force set point. In the transparency phase, the segment angles are used to calculate the amount of tendon length that should be reeled in or out by the motor-driving unit. An angular measurement error in this phase may result a too stiff of a human–robot interface, causing discomfort to the robot’s user. The reduction of the error achieved by the algorithm throughout both phases may thus lead to a better-timed assistive forces and improved control over the free tendon length in the transparency part of the algorithm.

### 4.3. The magnitude of the applied force affects the posture estimation error

The results of the linear mixed-effects model show a strong correlation between the average ( 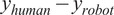 ) RMS error of the thigh segment angles and the used assistance force level, matching the observations shown by Langlois et al. (2021). This result is also in agreement with our expectations that a higher applied assistive force increasingly loads the human–robot interface leading to a higher displacement of the robotic components during the force on–off switching events. Depending on the particular architecture of a robot, the application of a higher force could either align or miss-align the robot segments with human segment, while the force relaxation would then play an opposite role to that.

The increase in the walking speed did not affect the averaged RMS error; the results at the assistance levels 1 and 5 were nearly identical for the two walking speeds. The linear trend was less prominent when comparing the different speed and assistance level results for the average ( 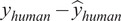 ) angle RMS error. Instead, the average RMS error increased when going from assistance level 1 to assistance level 3, but then flat-lined at the value of around 2.8° when the assistance level was further increased to level 5. This effect was observed for both walking speeds. These findings suggest that, for the considered experimental conditions, the model is capable of reducing the compliance-driven posture estimation error for all of the used force and speed values.

### 4.4. Additional considerations

As previously discussed, the inherent compliance of the human–robot interface across wearable robots means that posture estimation errors are present in most untethered wearable robotic devices, irrespective of whether the devices are soft (e.g., exosuits) or rigid-frame (e.g., exoskeletons) (Langlois et al., 2021).

With the exception of the marker point clouds, the selection of the features used for the model was limited to only those that can be expected to be available on most wearable robotic systems. This choice of the feature tree suggests that, given one has an accurate model of the sensor measurement error term, the proposed methodology can be applied to other wearable robotic devices and sensor setups with relative ease. Such an implementation would lead to a better timed force delivery for gait symmetry-based assistance profiles, as proposed by Malcolm et al. (2018) and Aguirre-Ollinger and Yu (2021). We believe that with the improved estimation of the segment angles the control algorithms of wearable robots can provide a more personalised magnitude and timing of the assistance forces. Such open source GitHub repositories as *m2cgen* provide further assistance in migrating the machine learning models from higher level languages (such as Python) to C, ultimately allowing for an embedded implementation.

Prior literature on various motion capture approaches suggest that the issue of relative motion between the user’s skin and the robot-mounted sensors can be partly reduced through custom-designed clothing combined with tight straps (Li et al., 2014; Mihcin et al., 2019). In addition, successful attempts to reduce the relative motion between wearable robots and their users have also been performed using custom-made orthoses that provide a larger contact area and leverage user-specific body landmarks at the points of robot’s force anchoring (Langlois et al., 2018). However, the relative motion between a robot and its user will always exist due to the soft, and thus compliant, nature of human tissue. Our algorithm partly accounts for the negative effects of such relative motion using a software-based solution only. This may be advantageous in some situations, as our approach does not depend on the manufacturing of user-specific robot components and does not affect the overall comfort of using a wearable device, as could be the case with an over-tightened strap.

### 4.5. Limitations and future work

Within the scope of this study, the relative motion between the robot and the human was quantified using camera-based motion capture systems. This stems from the attempt to isolate the segment estimation error due to the human–robot interface compliance from other types of errors as much as possible (e.g., inherent IMU sensor fusion algorithm error). It thus remains to be seen if the proposed method could be further extended to capture the errors related to both the compliance of the human–robot interface as well as the sensor integration algorithms.

When used on the surface of the lower limbs, motion capture markers may introduce inaccuracies in the angle estimations due to the soft-tissue artifacts. To counter such effects, we used additional markers, bony landmarks (whenever possible) and pre-compressed the tissue around the markers with strips of elastic tape. While the methods of quantification and minimisation of soft-tissue artifacts were outside of the scope of this project, an alternative marker cloud configuration (e.g., higher number of markers), may further increase the accuracy of posture estimation.

Recently published literature has demonstrated feasibility of using wearable robotics for a wide range of walking speeds, including running (Kim et al., 2019). When used in an outside environment or on a rough terrain, the walking speed of a robot’s user can vary significantly from one step to another. In this study, however, only constant walking speed profiles of 0.8 and 1.3 m/s, precisely controlled by using a constant-speed treadmill, were used. While we found no significant statistical dependency between the speed of walking and the magnitude of the compliance-driven error, it is not possible to claim with the limited acquired data that the two effects are fully independent for the full range of possible walking speeds that may be seen in an outside environment.

In this work, no attempt was made to define the required anthropometric parameters, such as the height, weight, and BMI, of the recruited participants. For this project, participants of wide height (1.62–1.95 m) and mass (51–85 kg) ranges were recruited. The Myosuit’s user manual, however, permits an even wider range of users: height between 1.5 and 1.95 m, body mass between 45 and 110 kg. It may be of benefit for the algorithm’s performance to select the participants such that the full range of parameters of the robot’s intended users are covered.

The fitting of the robot on the participants was neither controlled nor measured during the donning procedure. Instead, the Myosuit was donned following the procedures laid out in its instructions for use. While this is indeed the realistic use-case scenario it would be of interest to attempt to understand how the different fitting configurations affect the human–robot interface stiffness and ultimately the segment estimation error. We theorize that the effects of different strapping pressure were partly captured in the motion data of Myosuit’s IMU sensors at the time of force application and relaxation. Controlling and measuring the fitting would allow to further investigate this theory. Combining such measurements with a defined comfort score could additionally drive the guidance on robot donning procedures.

This study used a lower-limb exoskeleton for the proof of concept. As the errors in sagittal plane are often of most interest for lower-limb wearable robots, the angular errors in the other planes were not discussed. If this method were to be applied to an exoskeleton of a different configuration (e.g., upper extremity exoskeleton), the required compensation model may be more complicated and require additional feature engineering steps, such as the frontal and transverse plane components of the 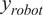 segment angle measurements.

Only the errors measured during level ground walking were considered in this study. Prior literature has shown that the angles of the lower limbs in uphill and downhill walking differ significantly from those in level ground walking (Nuckols et al., 2020). Within the domain of lower-limb assistance devices, including other activities (e.g., uphill and downhill walking, stair negotiation, and sitting transfers) can lead to a wider range of considered joint angles. This would further expand the relevance of the presented methodology to a bigger set of lower-limb wearable robots.

## 5. Conclusions

The importance of accurate estimation of a wearer’s posture is a known factor in the field of wearable robotics. In this study, we have shown that compliance-related errors that arise when wearable robot user’s posture is estimated based on the robot-mounted sensor array may be partially reduced by the use of machine learning algorithms. We have isolated the effects of compliance by using a camera-based motion capture system for both the human lower limbs segments and for the corresponding robotic segments. With our focus on the thigh segment, we have shown that the compliance effects have been most prominent in the regions between 40% and 80% of the gait cycle (with 0% defined as the heel strike). By combining the robot segment angle with other features derived from the robot’s sensors, a two-fold reduction of average RMS and an almost three-fold reduction in maximum RMS errors could be achieved.

In general, the compliance of the human–robot interface is a complex issue that depends on a number of factors, including, among others, the particular robot architecture, appropriateness of the robot’s fit on its user, and the mode of the applied assistive forces. In this project, we limited the number and the types of features used for the ML algorithm to those, that would be typically available on various robotic assistive devices. Here, the choice of the particular segment of interest and the focus on the sagittal plane were driven by the specific type of used lower-limb wearable robot. Future studies should limit the use of the camera-based motion capture to only measure the human segment angles, as well as investigate the extension of our proposed algorithm to other robot architectures and other planes of motion. Nevertheless, we believe that by following the procedures defined in this project, more personalised tailoring of the wearable robot controllers may be achieved, ultimately leading to a more individually targeted human assistance strategies, rehabilitation programs, and recovery progress reports.

## Data Availability

The data that support the findings of this study are available upon request from the corresponding author G.K.
